# Mirtazapine loaded polymeric micelles for rapid release tablet: A novel formulation—In vitro and in vivo studies

**DOI:** 10.1007/s13346-024-01525-w

**Published:** 2024-02-14

**Authors:** Sara Nageeb El-Helaly, Amira A. Rashad

**Affiliations:** 1 Department of Pharmaceutics and Industrial Pharmacy, Faculty of Pharmacy, Cairo University, Cairo, Egypt; 2 Department of Pharmaceutics and Pharmaceutical Technology, Faculty of Pharmacy, Heliopolis University, El Salam City, Cairo, Egypt

**Keywords:** Mirtazapine, Polymeric micelles, Rapid release tablet, Solubility enhancement, Pharmacokinetic study

## Abstract

Major depression is a prevalent disorder characterized by sadness, lack of interest or pleasure, interrupted sleep or food, and impaired concentration. Mirtazapine (MTZ), a tetracyclic antidepressant drug, is commonly used to treat moderate to severe depression. MTZ is classified as a BCS class II drug that has shown bioavailability of 50% due to extensive first-pass metabolism. The aim of this research is to develop a delivery platform with enhanced solubility and oral bioavailability of MTZ through formulating polymeric micelles modeled in a rapid release tablet. Mirtazapine loaded polymeric micelles (MTZ-PMs) were formulated to enhance the solubility. Solutol^®^ HS 15 and Brij 58 were used as combined surfactants in a ratio of (20:1) to MTZ in addition to Transcutol^®^ P as a penetration enhancer. The following in vitro tests were performed: particle size, PDI, zeta potential, solubility factor, stability index, and transmission electron microscopes. Afterward, MTZ-PMs were converted to dry free flowable powder through loading on the adsorptive surface of Aerosil 200; then, the powder mixture was directly compressed (MTZ-PMs-RRT) into 13 mm tablets. MTZ-PMs-RRT was further investigated using in vitro evaluation tests of the tablets, namely, weight variation, thickness, diameter, hardness, friability, disintegration time, drug content, and in vitro dissolution test, which complied with the pharmacopeial limits. The pharmacokinetic parameters of MTZ-PMs-RRT compared to Remeron^®^ tablet were further investigated in rabbits. The results showed enhanced solubility of MTZ with improved percentage relative bioavailability to 153%. The formulation of MTZ in the form of MTZ-PMs-RRT successfully improved the solubility, stability, and bioavailability of MTZ using a simple and scalable manufacturing process.

**Graphical Abstract:**

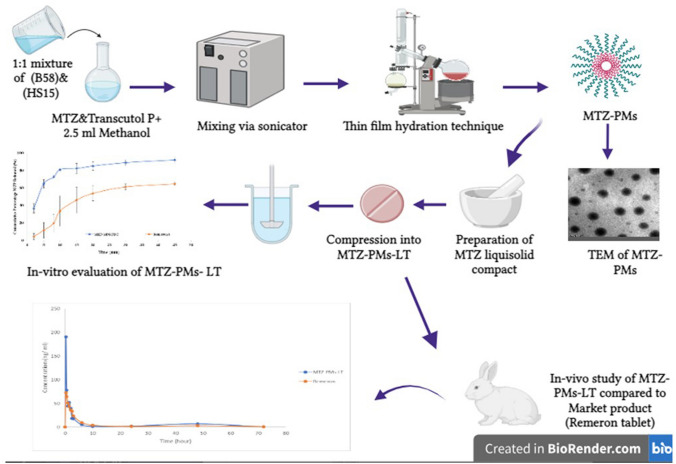

## Introduction

Major depression is a prevalent disorder that impairs psychosocial functioning and lowers quality of life. In 2008, the WHO listed major depression as the third leading cause of illness burden worldwide, with the disorder expected to rise to first place by 2030 [1]. Sadness, lack of interest or pleasure, interrupted sleep or food, feelings of weariness, and impaired concentration are all symptoms of depression. It can be long-lasting or recurring, significantly limiting a person’s ability to perform at work or school or cope with day-to-day activity leading at its most extreme to suicide. Depression frequently begins at an early age. It is affecting women more than men, and jobless individuals are more vulnerable as well [2]. Depression has been connected to brain disorders or imbalances involving the neurotransmitter’s serotonin, norepinephrine, and dopamine. According to research, measuring the level of neurotransmitter in a person’s brain is extremely challenging [3].

Mirtazapine (MTZ), one of the active compounds in the chemical class piperazinoazepines, is a tetracyclic antidepressant drug used to treat moderate to severe depression [4]. It works by antagonizing adrenergic alpha2-autoreceptors and alpha2-heteroreceptors, blocking 5-HT2 and 5-HT3 receptors, and increasing the release of norepinephrine and 5-HT1A-mediated serotonergic transmission [5, 6]. Mirtazapine’s effectiveness is hypothesized to be mediated in part by increased activation of the central 5-HT1A receptor. This combination of actions is thought to contribute to its effectiveness and quick onset of action [7].

MTZ is BCS class II with a long half-life (20–40 h) and has a bioavailability of 50% due to poor aqueous solubility, first-pass metabolism, and high protein binding (80%) [8, 9]. Following oral administration, mirtazapine reaches peak plasma concentration approximately in 2 h [4]. Mirtazapine is available only as tablets in doses of 15, 30, and 45 mg, respectively. Several research articles worked on improving MTZ properties like formulation of MTZ as nanoemulsion, chitosan microspheres, PLGA microparticles, and orally disintegrating tablets [10].

In recent times, the combination of two or more surfactants to form polymeric micellar systems has attracted the attention of researchers as a promising nanotechnology-based drug delivery approach to improve drug solubility [11]. They are formed when the concentration of hydrophilic and lipophilic chains in water surpasses a particular threshold known as the critical micelle concentration (CMC). The inner core is hydrophobic and can entrap hydrophobic drugs, whereas the shell is hydrophilic, which can stabilize the preparation in an external aqueous solution [12]. Because of their inherent and customizable properties, they are especially well-suited for drug delivery. Polymeric micelles have several benefits, including the ability to solubilize poorly soluble compounds, provide prolonged release and size advantages, and shield encapsulated compounds from degradation and metabolism. As a result, polymeric micelles are highly adopted for drug delivery of challenging molecules [13]. Despite the potential benefits, the application of micelles in drug delivery is limited due to factors such as poor loading capacity, inadequate physical stability in vivo, and the absence of suitable methods for scaling up, especially when considering their liquid nature [14]. These limitations need to be overcome through using of additional technique aiming to make the polymeric micelles more applicable in pharmaceutical industry.

Our study is aimed at enhancing the solubility and bioavailability of MTZ through formulating MTZ loaded polymeric micelles as a liquid system into a solid oral dosage form. Mirtazapine will be incorporated into polymeric micelles to minimize particle size reaching nano size, enhance the solubility, and bypass the first metabolism. In addition, such a formulation will be converted to dry free flowing powder through loading of MTZ-PMs on the adsorptive surface of Aerosil 200 to stabilize the formed polymeric micelles; then, the powder will be directly compressed into rapid release tablet (MTZ-PMs-RRT).

## Materials and methods

### Materials

Mirtazapine was purchased from Sinochem, China. Solutol (HS15) and BrijVR 58 (B58) (polyethylene glycol hexadecyl ether) were obtained from Sigma-Aldrich Inc., USA. Transcutol P (Trc) (diethylene glycol monoethyl ether) was supplied as a gift by Gattefosse, France. Mannitol was obtained from Shandong Bangye Co. Ltd., China. Explotab (sodium starch glycolate) was obtained from JRS Pharma LP., USA. Magnesium stearate was obtained from Aceto-Corp, USA. Aerosil 200 (colloidal silicon dioxide) was purchased from Degussa Ltd., Germany. Methanol (95%), disodium phosphate hydrogen, and potassium dihydrogen phosphate were obtained from El-Nasr Pharmaceutical Chemicals Co., Egypt. All other solvents and chemicals were of analytical grade. The water used was distilled, deionized water.

### Animals

Sixteen male albino rabbit were used in this study. The study described was approved by the Research Ethics Committee (REC) at the Faculty of Pharmacy, Cairo University, with the approval number (PI 3412). The study was conducted in compliance with the guidelines provided in the “Guide for Care and Use of Laboratory Animals” published by the US National Institute of Health [15].

### Preparation of mirtazapine loaded polymeric micelles (MTZ-PMs)

Mirtazapine polymeric micelles were prepared by thin-film hydration method [16], and the compositions are shown in Table 1. In brief, based on optimized study in multiple research articles [12, 17], a binary 1:1 mixture of surfactants (B58) and (HS15) were added to MTZ (15 mg) at (20:1) ratio along with 5 mg Transcutol P as a penetration enhancer. All the components were co-dissolved in methanol (2.5 mL) using the ultrasonic method in a 500 mL round bottom flask. The solvent was then evaporated under reduced pressure using a rotary vacuum evaporator (Heidolph VV 2000, Burladingen, Germany) at 60 °C and 150 rpm until a thin dry film formed on the walls of the flask. Next, the dried film was hydrated by adding 5 mL of distilled water under normal pressure at 60 °C and 150 rpm for 30 min. The resulting mirtazapine polymeric micelles were then stored in a refrigerator at a temperature of 8 ± 2 °C for further investigation [17].

**Table 1:** Composition of MTZ loaded polymeric micelles and MTZ-PMs-RRT

**Components**	**Amount/1 mL PM**	**Components**	**Amount/800 mg tablet**
**Mirtazapine**	15 mg	**Polymeric micelles**	0.5 mL (500 mg), equivalent to 7.5 mg mirtazapine
**Brij58**^**a**^	150 mg	**Aerosil 200**	160 mg
**Solutol**^**a**^	150 mg	**Explotab**	64 mg
**Transcutol P**	5 mg	**Mannitol**	68 mg
**Methanol**	0.5 mL	**Mg stearate**	8 mg
**Water**	To 1 mL	**Total weight**	800 mg

^a^Brij58 and Solutol were added in a 1:1 ratio to make surfactants to mirtazapine ratio of (20:1)

### Characterization of the prepared mirtazapine loaded polymeric micelles

#### Determination of particle size (PS), polydispersity index (PDI), and zeta potential (ZP)

The mean particle size, size distribution, and polydispersity index (PDI) of the polymeric micelles were determined at 25 °C using dynamic light scattering with a Malvern Zetasizer (Nano ZS; Malvern Instruments, Worcestershire, UK). The analysis was performed three times to obtain the mean values. Each analysis lasted for 60 s at an angle detection of 175°. The micelles were also characterized for zeta potential using the same instrument. The samples were diluted in deionized water and measured in triplicates using disposable measurement cells [18].

#### Solubility factor (Fs) and stability index (SI)

To determine the enhancement of MTZ solubility (solubility factor), freshly prepared polymeric micelles containing MTZ were filtered through a 0.45 µm nylon membrane filter to remove insolubilized MTZ obtaining clear mirtazapine loaded polymeric micelles (MTZ-PMs). A 100 µL sample was diluted to 10 mL with absolute ethanol, and the amount of MTZ solubilized was determined spectrophotometrically at λ-max 292 nm with a UV-1900 spectrophotometer (Shimadzu Europe GmbH, Germany) and compared to the reported MTZ aqueous solubility. The same procedure was repeated after 48 h to measure the amount that is still soluble. The stability index is a measure of polymeric micelle capability to maintain the solubilized amounts of MTZ and hence stability of the formulation. The solubility factor (Fs) and stability index (SI) were calculated using the following equations:

$${F}_{S}=\frac{{S}_{M}}{{S}_{W}}$$

$$\textit{SI}=1-\left(\frac{S_T-S_M}{S_T}\right)$$

where *S*_*M*_ is the solubility of MTZ in PMs after 48 h, *S*_*W*_ is the reported aqueous solubility of MTZ in water, and *S*_*T*_ is the solubility of MTZ in freshly prepared PMs [17].

#### Transmission electron microscopy (TEM)

For further analysis, one drop of the diluted MTZ-PM sample was placed onto a carbon-coated copper grid. To negatively stain the sample, one drop of a 2% w/v phosphotungstic acid solution was added. After allowing the samples to air dry, they were examined using a transmission electron microscope (JEM-1230; JEOL, Tokyo, Japan) operating at 80 kV [17].

### Preparation of MTZ-PM rapid release tablets

The composition of MTZ-PMs rapid release tablets was shown in Table 1. Briefly, the preparation method started by loading 0.5 mL of MTZ-PMs on the adsorptive surface of Aerosil 200 to absorb surplus liquid and give the MTZ-PMs a dry appearance and increase their flowability. Then, further excipients were added to the formulation to enhance dissolution and induce compression, Explotab, a super disintegrant, and mannitol as a diluent [19]. These excipients, along with the previously mentioned components, were geometrically mixed in a mortar for 20 min. The resulting mixture was then passed through a sieve with a 500 μm mesh size. To further blend the mixture, 1% magnesium stearate was added and combined for 2 min. The final blend was compressed into 800 mg tablets using a single punch tablet machine (KORSCH XP1) with a 13-mm round diameter punch [20].

### Characterization of the prepared of MTZ-PM rapid release tablets (MTZ-PMs-RRT)

#### Post-compression evaluation of the tablet

Weight variation was measured with universal balance AUY 220, Sartorius, Germany. Thickness, diameter, and hardness were measured with vernier caliper and tablet hardness tester (Copley TH3/200 Scientific Limited, UK). The friability test was conducted using friability tester (Copley FR1000, UK) at 25 rpm speed, and the percent of weight loss was calculated. Disintegration test was performed with disintegration test machine (Copley DTG 1000, UK). In addition, in vitro drug release study was performed using dissolution apparatus (Copley DIS 6000, UK) adopting the method described in USP dissolution database [21].

#### Determination of drug content

In order to determine the MTZ content in the tablets, ten randomly selected tablets were powdered using a mortar and pestle. From this powder, an amount equivalent to 7.5 mg of MTZ was transferred into a 10 mL volumetric flask. To this flask, 5 mL of methanol was added, and the mixture was shaken for 15 min. The volume was then made up to 10 mL with methanol. The resulting solution was filtered, and 1 mL of the filtrate was diluted and analyzed using a UV-spectrophotometer (1650 PC, Shimadzu, Japan) at a wavelength of 292 nm (λ-max). The MTZ content was calculated using a prepared calibration curve.

#### In vitro dissolution studies

**Fig. 1:**
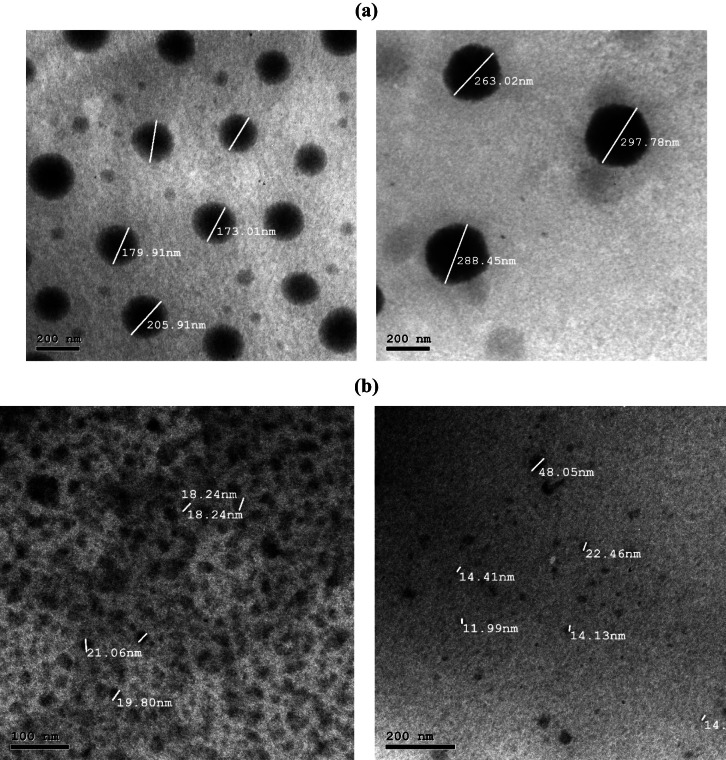
Transmission electron micrographs of mirtazapine loaded polymeric micelles (MTZ-PMs): **a** *Z*-average particle size of MTZ-PMs and **b** particle size at 68.9% intensity

The dissolution test was performed according to the method specified in USP dissolution database. In this method, the USP type II dissolution test apparatus (Copley DIS 6000, UK) was used at 37 ± 2 °C and 50 rpm. A total of 250 mL of 0.1N hydrochloric acid was used as dissolution medium. Six tablets were examined for each MTZ-PMs-RRT and Remeron^®^ 30 mg tablets as reference market product. An aliquot equal to 5 mL was withdrawn at 2, 5, 10, 15, 20, 30, and 45 min [22] and replaced with 5 mL fresh preheated dissolution medium. The collected samples were filtrated, and the cumulative % release of MTZ in MTZ-PMs-RRT and Remeron^®^ tablet was determined spectrophotometrically at λ-max 292 nm [21]. Finally, the mean dissolution percentage and SD were calculated. The results of drug dissolution are depicted in Fig. 1 [21, 23].

### In vivo pharmacokinetic study

#### Study design

The in vivo studies were conducted in accordance with EU Directive 2010/63/EU for animal experiments. The pharmacokinetics of MTZ from MTZ-PMs-RRT was compared to the commercial reference tablet “Remeron^®^ 30 mg” using a non-blind, randomized, two-treatment, two-period, two-sequence, and crossover design. The study took place in the Animal House of the Faculty of Pharmacy, Cairo University. Sixteen male white albino rabbits weighing between 1800 and 2000 gm were used for the study. They were housed in optimal environmental conditions, including a room temperature of 25 °C, humidity of 60 ± 10%, and a 12-h light and dark cycle. The rabbits fasted for 10 h prior to the study and continued to fast for 4 h after receiving the drug, although they had access to drinking water [24]. The study was conducted in accordance with the guidelines of the Cairo University, Faculty of Pharmacy, Institutional Animal Care and Use Committee, with the approval number (PI 3412).

#### Determination of MTZ concentration in rabbit plasma

The study was conducted in two phases: phase I, sixteen rabbits were randomly divided into two groups. Group I received MTZ-PMs-RRT at a dose equal to 7.5 mg of MTZ, and group II was given Remeron^®^ 30 mg. Administration was orally by pill dispenser feeding kit. Blood samples (0.5 mL) from ear vein were collected at 0, 0.25, 0.5, 1, 1.5, 2, 2.5, 3, 6, 10, 24, 48, and 72 h in a heparinized tube, centrifuged at 4000 rpm for 15 min (min) to separate plasma. A washout period of 8 days separated the two phases. On the second phase, the reverse of randomization was performed [25]. Plasma was kept at − 20 °C until plasma extraction recovery of MTZ using vacuum evaporation techniques was performed. Plasma concentrations of MTZ were analyzed using LC/MS/MS (UPLC system-Triple Quad 4500, Shimadzu LC Technologies, Japan). The mobile phase was composed of 80% acetonitrile + 20% H_2_O + 0.1% formic acid, and internal standard was 10 Ug/mL bupropion. Then, 5 μL aliquots of the samples were injected at flow rate of 0.8 mL/min and purge time of 25 min. The chromatographic separation was carried out using isocratic flow on Waters Sunfire C18 column (4.6*50 mm), 5µm.

#### Calculation of pharmacokinetic parameters

The principal pharmacokinetic parameters, including maximum plasma concentration (*C*_max_), time to reach *C*_max_ (*T*_max_), area under the plasma concentration–time curve (AUC_0-*t*_ and AUC_0-∞_), and elimination half-life (*t*_1/2_), were calculated using Phoenix^®^ Winnonlin^®^ version 8.4. The results were expressed as the mean of 16 rabbits ± standard deviation (SD). To determine the relative bioavailability of the optimized MTZ-PMs-RRT compared to the Remeron^®^ 30 mg tablet, the following equation [26]:

$$\%\;\mathrm R\mathrm e\mathrm l\mathrm a\mathrm t\mathrm i\mathrm v\mathrm e\;\mathrm b\mathrm i\mathrm o\mathrm a\mathrm v\mathrm a\mathrm i\mathrm l\mathrm a\mathrm b\mathrm i\mathrm l\mathrm i\mathrm t\mathrm y=\frac{{\textit{AUC}}_\textit{test}\mathit\,\times\,{\textit{Dose}}_\textit{Reference}}{{\textit{AUC}}_\textit{Reference}\mathit\,\times\,{\textit{Dose}}_\textit{Test}}$$

To compare the pharmacokinetic parameters, *C*_max_, AUC_(0–24)_, and AUC_(0–∞)_, a student *T*-test was performed using SPSS Version 19. The nonparametric signed rank test (Mann–Whitney’s test) was used to compare mean residence time (MRT) and *T*_max_. A *p* value ≤ 0.05 was considered statistically significant.

## Result and discussion

### Preparation of mirtazapine loaded polymeric mixed micelles (MTZ-PMs)

One vital characteristic of mixed micelle systems is their capacity to dissolve medications that are poorly soluble. In our study, we combined MTZ with a binary mixture of amphiphilic surfactants B58 and HS15 in a ratio of 20:1. The amphiphilic properties of B58 have proven the ability to provide a high degree of solubilization for poorly water-soluble medicine (MTZ) based on several optimized tests published in multiple research articles [27]. This might be attributable to the structure’s low number of polyethylene oxide PEO units (PEO = 20) [28]. These PEO units produced a thick hydrophilic corona that protected the core from the entry of hydrophobic MTZ [29].

Younes et al. reported that the use of such two types of surfactants (HS15/B58) strengthened the hydrophobic contacts between their hydrophobic segments, which directly lowered the critical micelle concentration (CMC) of the resulting combination to 0.0039% w/v [17]. These hydrophobic interactions play a crucial role in maintaining the integrity of the micelle shell and preventing dissociation into individual monomers [30]. Duan et al. found that spontaneously generated micelles can have superior solubilization capabilities and optimal stability [31]. The current results found that an increase in the quantity of the mixed micelles led to better solubilization of MTZ. However, increasing the amount of drug relative to total surfactants may have resulted in rapid saturation of the mixed micelle's inner cores, causing the precipitation of excess drug. These results come in agreement with the findings reported by Fares et al. [16]. Transcutol P (Trc) is a co-surfactant in polymeric mixed micelles, due to its hydrophilic nature and miscibility with water, which enhanced the dissolution of poorly water soluble drugs as reported by Akl et al. and Chen et al. [32, 33]. Accordingly, Transcutol P was the cosurfactant of choice with MTZ-PMs.

### Characterization of the prepared mirtazapine loaded polymeric micelles (MTZ-PMs)

#### Determination of particle size (PS), polydispersity index (PDI), and zeta potential (ZP)

As shown in Table 2, the *Z*-average particle size of MTZ-PMs was 298.75 ± 131.87 nm, and the particle size at 68.9% intensity was 9.809 ± 0.467 nm. This small size can be attributed to the previously documented strong hydrophobic interactions between B58 and HS15, resulting in compact spherical micelles [33]. Moreover, the high surfactant-to-drug ratio (20:1) led to the formation of a large number of polymeric micelles that could effectively incorporate MTZ into their core without significant core swelling. These results run with that published by Younes et al. who used two surfactant-to-drug ratios (10:1) and (20:1) and found that the optimum ratio was (20:1) [17]. Surfactants also contribute to increased steric resistance by forming an adsorption layer on the particle surface, which leads to a decrease in particle size [34]. Such findings come in agreement with the data of Miyazawa et al. who announced surfactants play a critical role in the size of nanoparticles [35].

**Table 2:** In vitro characterization of mirtazapine loaded polymeric micelles (MTZ-PMs) and MTZ-PM rapid release tablets (MTZ-PMs-RRT)

**MTZ-PMs**	***Z*-average (nm)**	**PS (nm)**	**% intensity**	**PDI**	**ZP (mV)**	**Fs**	**SI**
	298.75 ± 131.87	9.809 ± 0.467	68.9	0.447 ± 0.171	3.075 ± 1.49	166.25 ± 1.33	1.09 ± 0.05﻿
**MTZ-PMs-RRT**	**Average weight (mg)**	**Thickness (mm)**	**Diameter (mm)**	**Hardness (Kg)**	**Friability**	**Disintegration (s)**	**Drug Content (%)**
	802.47 ± 18.65	13.21 ± 0.04	7.52 ± 0.17	3.6 ± 0.24	0.34 ± 0.17	34 ± 6.09	99.78 ± 3.7

*PS particle size, *PDI* polydispersity index, *ZP* zeta potential, *Fs* solubility factor, *SI* stability index

The polydispersity index (PDI) reflects the breadth of the particle size distribution. A PDI value closer to zero indicates a relatively homogeneous particle size distribution, while a value closer to one indicates a highly polydispersed distribution [36]. The average PDI value of 0.447 ± 0.171 indicates that the MTZ-PMs had a homogeneous particle dispersion.

Zeta potential (ZP) measures the net charge accumulated on the surface of the dispersed polymeric micelles. The magnitude of the ZP is indicative of the physical stability of the polymeric micelles. The ZP of the MTZ-PMs was found to be 3.075 ± 1.49 mV. The presence of cationic nitrogen atoms in the MTZ structure conferred a positive charge, which dominated over the neutral charge of the nonionic surfactants used, resulting in a positive ZP value. These findings were supported by Qushawy et al. who prepared miconazole nitrate nano-transfersomes and found that the prepared vesicles had positive zeta potential values due to the presence of cationic nitrogen atoms in the structure of miconazole nitrate [37]. The obtained results are in harmony with that detected by Zhen et al. who demonstrated that positively charged nanocomposite of curcumin could maintain long-term stability [38].

#### Solubility factor (Fs) and stability index (SI)

According to the literature, the solubility of MTZ (SM) in water is 0.092 mg/mL [26]. In terms of computed Fs, there is a 166-fold increase in solubility (166.25 ± 1.33). This might be owing to the surfactants’ solubilizing ability, which was demonstrated at high surfactant-to-drug ratios (20:1), resulting in greater micelle production and, thus, higher MTZ solubility, which coincides with that obtained by Shubber et al. [39]. The computed stability index (SI) values confirmed this assumption, revealing that MTZ-PMs had a high SI value of 1.09 ± 0.053. This meant that MTZ-PMs were able to sustain their assembled structure after 48 h and hence kept MTZ solubilized within their core. The solubility rose after standing to be greater than the initial value of the freshly created MTZ-PMs, implying that the prepared micelle’s stability increased with time.

#### Transmission electron microscopy (TEM)

The obtained PS values from the Zetasizer analyzer were further validated through TEM imaging [40]. The MTZ-PMs exhibited a spherical morphology, with a narrow size range and no evident signs of significant aggregation. This lack of aggregation could be attributed to the presence of electrostatic and steric barriers between the formed PMs, as illustrated in Fig. 1. Additionally, comparable results were introduced by Abbad et al. who declared that the mean particle size observed in the TEM micrographs concurred with the PS obtained from the Zetasizer analyzer [14].

### Characterization of the prepared of MTZ-PM rapid release tablets

#### Post-compression evaluation of the tablet

Table 2 shows the MTZ-PMs-RRT assessment findings. The friability of all tablets is 0.34% ± 0.17, indicating high mechanical strength and tolerance to physical handling conditions. The hardness of MTZ tablets is 3.6 ± 0.24 kg. None of the produced tablets had a hardness less than 3 kg. These values are within the preferred range of 2–8 kg, as suggested by Eisa et al. [41], which gives enough mechanical strength, porosity for disintegration, and wetting time. The prepared MTZ-PMS-RRT has thickness and diameter values of 7.52 ± 0.17 and 13.21 ± 0.04, respectively. Die fill uniformity, adequate flow characteristics, and powder compressibility were verified by tablet thickness and diameter values [42]. The MTZ tablets fulfilled pharmacopeial criteria with an average percentage of MTZ content of 99.78% ± 3.7 [43]. Weight variation range was 802.47 ± 18.65 mg. Disintegration time was 34 ± 6.09 s, showing that Explotab had a super disintegrating effect. These data are parallel to that achieved by Bishal et al. who indicated that Explotab was shown to have the best disintegrating and dissolution performances [44]. All in vitro characterization tests were within the pharmacopeial limits.

#### In vitro dissolution studies

**Fig. 2:**
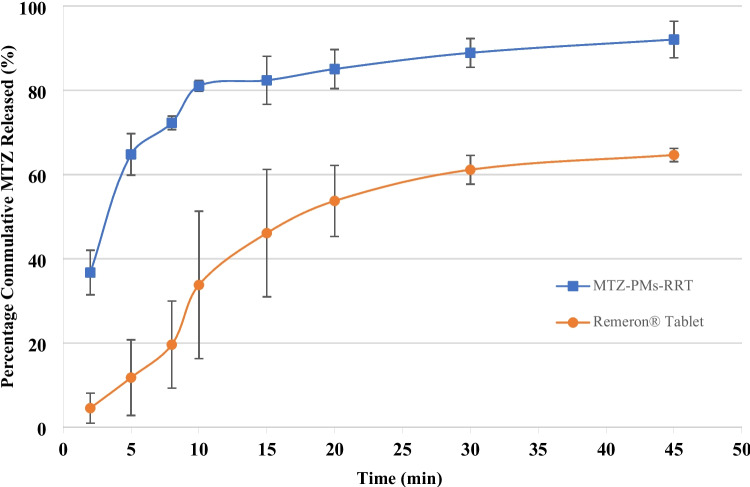
In vitro dissolution profile of MTZ from MTZ-PMs-RRT; mirtazapine loaded polymeric micelles in rapid release tablet compared to the market product; Remeron^®^ tablet in 0.1 N HCl at 37 ± 0.5 °C and 50 rpm

The prepared MTZ-PMs-RRT was compared to the market product Remeron^®^ 30 mg tablet. The plots of cumulative percentage releases of MTZ related as a function of time (t) for MTZ-PMs-RRT and Remeron^®^ 30 mg tablets were shown in Fig. 2. MTZ-PMs-RRT showed rapid drug release, 64.76% ± 4.93 after 5 min, compared to 11.77% ± 9 of MTZ release from Remeron^®^ tablet. The fast dissolution rate from MTZ-PMs-RRT might be due to rapid disintegration of the tablets to form particles and enhancing solubility of MTZ to about 166-folds which was observed previously from the results of solubility factor test and disintegration test. Such an enhancement may be due to decreased particle size, increased surface area, and micellar effect of both Brij58 and Solutol. This is comparable to that achieved by Ozturk-Atar et al. for improving dissolution behavior of poorly water-soluble tamoxifen citrate [18]. After 45 min, the release of MTZ from MTZ-PMs-RRT reached 92.06% ± 4.31, compared to 64.65% ± 1.57 from Remeron^®^ tablets.

### In vivo pharmacokinetic study

Pharmacokinetic parameters for MTZ in rabbits’ plasma were assessed using UPLC-MS/MS method in order to understand the in vivo behavior of the MTZ-PMs-RRT compared to the reference Remeron^®^ tablets. MTZ showed an absolute bioavailability after oral administration of only 50% due to poor aqueous solubility and first-pass metabolism [9]. Hence, we aimed to develop a micellar MTZ formulation with better in vivo performance than a drug formulation currently employed in the market.

**Fig. 3:**
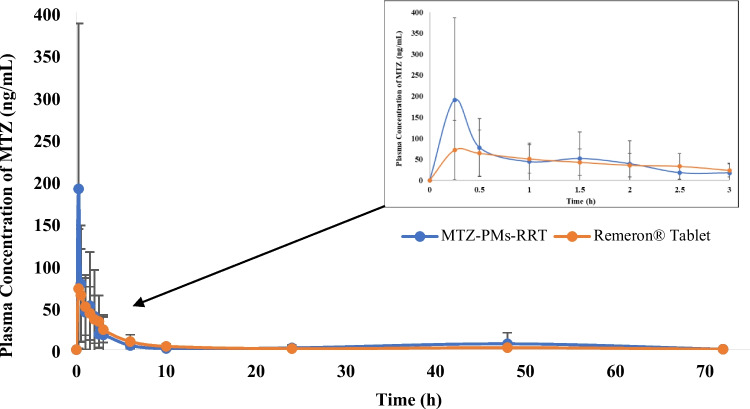
Mean plasma concentration time data after single oral administration of MTZ-PMs-RRT; mirtazapine loaded polymeric micelles in rapid release tablet compared to the market product; Remeron^®^ tablet

The values and the statistical analysis of the mean pharmacokinetic parameters obtained by noncompartmental fitting of the concentration–time data of MTZ-PMs-RRT and Remeron^®^ tablet are given in Tables 3, 4, 5, and 6, respectively. The plasma concentration time curve is shown in Fig. 3. After administration of MTZ-PMs-RRT, the concentration of MTZ was raised rapidly which was significantly higher than that obtained from Remeron^®^ tablets, where the mean *C*_max_/dose was 34.67 ± 35.68 ng/mL compared to 15.88 ± 11.19 ng/mL of Remeron^®^. The *C*_max_ attained after short *T*_max_ of 0.25 ± 0 h is earlier than that in Remeron^®^ tablet (0.875 ± 0.82 h). The higher *C*_max_ observed for MTZ-PMs-RRT suggests that a larger amount of MTZ was absorbed in the gastrointestinal (GI) tract compared to MTZ in the conventional market product Remeron^®^ tablet. This difference in absorption could be attributed to the contrasting in vivo behaviors of the two formulations. In the case of Remeron^®^ tablet, the hydrophobic nature of MTZ made it difficult for the drug to be absorbed efficiently, leading to reduced accumulation in the bloodstream and a lower *C*_max_. In contrast, MTZ-PMs improved drug solubility and stability in the GI tract due to the presence of micelles. Additionally, these findings are supported by Lu et al. who stated that the small particle size enabled the micelles to penetrate through the pre-epithelial layer of mucus and be taken up by enterocytes [45]. Furthermore, free MTZ, after the diffusion of the micelles, could enter enterocytes with the assistance of various surfactants and be absorbed through passive diffusion [46]. As a result, MTZ-PMs significantly increased the total MTZ concentration in the plasma and greatly improved the oral bioavailability of MTZ.

**Table 3:** Mean pharmacokinetic parameters of MTZ-PMs-RRT and market product; Remeron^®^ tablets

**Pharmacokinetic parameters**	**Remeron**^**®**^** tablet**	**MTZ-PMs-RRT**
***C***_**max**_**/dose**	15.88 ± 11.19	34.67 ± 35.68
***T***_**max**_	0.875 ± 0.82	0.25 ± 0
**AUC**_**(0–72)**_**/dose**	58.43 ± 51.75	77.55 ± 64.71
**AUC**_**(0–∞)**_**/dose**	60.11 ± 52.8	82.29 ± 70.101
***T***_**1/2**_	9.3 ± 4.23	21.15 ± 16.05
**MRT**	11.99 ± 7.47	19.2 ± 20.05
***K***_**el**_	0.098 ± 0.073	0.046 ± 0.027
**% relative bioavailability**	**153%**	

Values in bold highlight supremacy of the MTZ-PMs-RRT over the market product

**Table 4:** Statistical analysis of mean pharmacokinetic parameters of MTZ-PMs-RRT and market product; Remeron^®^ tablets

**One-sample test**						
Test value = 0						
	*t*	df	Sig. (2-tailed)	Mean difference	95% confidence interval of the difference	
					Lower	Upper
C_max__D	3.955	15	0.001	25.2797711	11.656027	38.903515
Lambda_*z*	5.183	15	0.000	0.0728690	0.042904	0.102834
Halflife	5.012	15	0.000	15.2303232	8.753364	21.707282
AUClast_D	5.100	15	0.000	67.9936251	39.576065	96.411186
AUCinf_D	5.025	15	0.000	71.2038465	41.003673	101.404020

**Table 5:** Non-parametric Mann-Whitney test of mean *T*_max_

**Independent-samples Mann–Whitney *U* test summary**	
Total *N*	16
Mann–Whitney *U*	16.000
Wilcoxon *W*	52.000
Test statistic	16.000
Standard error	7.230
Standardized test statistic	− 2.213
Asymptotic Sig. (2-sided test)	0.027
Exact Sig. (2-sided test)	0.105

**Table 6:** Non-parametric Mann-Whitney test of mean MRT

**Independent-samples Mann–Whitney *U* test summary**	
Total *N*	16
Mann–Whitney *U*	36.000
Wilcoxon *W*	72.000
Test statistic	36.000
Standard error	9.466
Standardized test statistic	0.423
Asymptotic Sig. (2-sided test)	0.673
Exact Sig. (2-sided test)	0.721

These findings indicate that MTZ-PMs not only enhance the solubility of MTZ but also improve its permeability through intestinal epithelial cells, leading to a significantly shorter *T*_max_, higher *C*_max_, and improved bioavailability. The current results support the report of Chen et al. who claimed that PMs is a promising way for improving the curcumin oral bioavailability [47].

The differences between the two treatments for *C*_max_/dose and *T*_max_ were found to be statistically significantly different (*p* = 0.001 and 0.027, respectively). The non-parametric signed rank test (Mann–Whitney’s test) showed a significant difference between *T*_max_ of both treatments. The significant prolongation in the *T*_1/2_ (*p* value 0.000) and insignificant MRT (*p* value 0.673) in the case of MTZ-PMs-RRT (21.15 ± 16.05 h and 19.2 ± 20.05 h), respectively, compared to that of Remeron^®^ tablet (9.3 ± 4.23 h and 11.99 ± 7.47 h), could be attributed to the encapsulation of MTZ in the core of micelles. The hydrophobic core structure of the micelles delays the diffusion of water into the core, leading to a prolongation in the elimination half-life (*T*_1/2_). This property prevents rapid leakage and precipitation within the gastrointestinal (GI) tract during oral drug delivery [48].

Regarding the extent of drug absorption as indicated by the AUC_(0–∞)_ value, significantly higher value was obtained from MTZ-PMs-RRT, where the mean AUC_(0–∞)_/dose was found to be 82.29 ± 70.101 ng.h/mL, which was 1.36-fold higher and statistically significantly different (*p* = 0.000) than Remeron^®^ tablets (60.11 ± 52.8 ng.h/mL). The percentage relative bioavailability (%RB) was found to be 153%. This can be attributed to the incorporation of MTZ into the micellar nanoformulation, which helps bypass extensive first-pass metabolism. The micellar system also reduces drug metabolism and elimination as the pharmacokinetic properties of the drug are modified to those of the micelles. The nonionic nature of the polymers in the formulation decreases electrostatic repulsion between the drug molecules, resulting in a hydrophilic shell formation around the core. This significantly increases the solubility and stability of the formulation. These findings are in a close agreement with that presented by Ashwini et al. on using PMs of sulphasalazine for improvement of oral bioavailability [49].

## Conclusions

This research is aimed at enhancing the solubility and bioavailability of a BCS class II drug (MTZ) using polymeric micelles system and formulating this liquid system into a solid oral dosage form. The polymeric micelles formed were reproduced successfully based on a chosen ratio (20:1 mixed surfactant to drug ratio) from literature review that was hypothesized to produce stable micelles with high solubility factor. MTZ-PMs were loaded over the adsorptive surface of Aerosil 200 to form a dry free-flowing powder. This technique maintained MTZ solubilized in polymeric micelles system without alternative complicated drying methods. The dry powder was then mixed with the proper excipients for direct compression into a rapid release tablet (MTZ-PMs-RRT) which demonstrated post-compression evaluation tests within the acceptable pharmacopeial limits. MTZ-PMs-RRT showed rapid release profile compared to market product Remeron^®^ tablet indicating the successful improvement in the dissolution rate of MTZ. These results were further confirmed by the in vivo pharmacokinetic study of MTZ-PMs-RRT against Remeron^®^ tablet which showed enhancement in the percentage relative bioavailability. Based on the findings, the novelty of this research work lies in developing a simple and scalable method for the pharmaceutical industry to formulate polymeric micelles into an oral solid dosage form with high physical stability and enhanced bioavailability.

## Data Availability

The datasets generated during and/or analyzed during the current study are available in the supplementary material file submitted and from the corresponding author on reasonable request. The datasets generated during and/or analyzed during the current study are available from the corresponding author on reasonable request.
